# Association between Disability and Unmet Food Needs in the Venezuelan Migrant and Refugee Population: Analysis of a Population-Based Survey, 2022

**DOI:** 10.3390/nu15071663

**Published:** 2023-03-29

**Authors:** Akram Hernández-Vásquez, Alicia Bartra Reátegui, Keller Sánchez-Dávila, Rodrigo Vargas-Fernández

**Affiliations:** 1 Centro de Excelencia en Investigaciones Económicas y Sociales en Salud, Vicerrectorado de Investigación, Universidad San Ignacio de Loyola, Lima 15046, Peru; 2 Vicerrectorado de Investigación, Universidad Nacional de San Martín, Tarapoto 22201, Peru; abartra@unsm.edu.pe (A.B.R.); ksanchezd@unsm.edu.pe (K.S.-D.); 3 Faculty of Health Sciences, Universidad Científica del Sur, Lima 15067, Peru; jvargasf@cientifica.edu.pe

**Keywords:** immigrants, refugees, food, people with disability, Peru

## Abstract

In Peru, Venezuelan migrants and refugees have been exposed to food shortages before their emigration. This problem could have worse outcomes in vulnerable populations (such as people with disabilities); however, the literature on the basic needs of this population is still scarce. The objective was to determine the association between the presence of disability and the unmet need for access to food in the household of the Venezuelan migrant and refugee population residing in Peru. A cross-sectional study was conducted using data from the Second Survey of the Venezuelan Population Residing in Peru (ENPOVE 2022). The outcome variable was unmet need for food, while the independent variable was the presence of disability. Poisson log generalized linear regression models (crude and adjusted for potential confounding variables) were fitted to evaluate the association between the variables of interest, reporting prevalence ratios (PR) and 95% confidence intervals (CIs). A total of 7739 migrants and refugees from Venezuela were included. The proportion of unmet need for access to food in the household was 45.2%, while the proportion of disability was 2.1%. People with disabilities were found to be more likely to have an unmet need for access to food at home (adjusted PR [aPR]: 1.25; 95% CI: 1.08–1.46; *p* = 0.003). According to our findings, almost half of Venezuelan households were found to have an unmet need for access to food. In addition, Venezuelan migrants and refugees with disabilities were more likely to have an unmet need for this basic need.

## 1. Introduction

People with disabilities are a vulnerable and underserved group who experience difficulties in accessing health, education, employment, and social support services [1]. It is estimated that more than one billion people around the world live with disabilities (representing 15% of the world’s population) [1,2], and about 80% of this population resides in low- and middle-income countries (LMIC) [3]. In Latin America and the Caribbean (LAC), the highest proportion of migrants and refugees with disabilities and their families reside in Peru (31.5%), followed by Colombia (15.1%) and the Dominican Republic (11.8%). Forty percent are male, aged 36 to 59 years (43.2%), and they are illegal immigrants in the city where they reside (53.8%) [4]. Despite the fact that government institutions seek to ensure that people with disabilities have access to health services, education, employment, and other basic needs, this population experiences worse health outcomes and inequalities in access to food compared to the general population, which could be further accentuated by their migratory status [1,5]. However, data on the proportion of migrants and refugee population are scarce and vary considerably from one region to another [6,7], which could be attributed to the lack of recognition of forms of disability; lack of uniform definitions of disability; and cultural, social and economic barriers that hinder the migration of people with disabilities.

In LAC, the Venezuelan migrant and refugee population have maintained an unsatisfied need for food since before their emigration, because in their cities of origin, they experienced food shortages and high costs, demonstrated by a hypocaloric diet low in nutrients [8]. In addition, in LMIC (as in the countries that make up the LAC region), people with disabilities live in conditions of poverty and extreme poverty, with a lack of access to health services, inadequate sources of drinking water and sanitation, and a lack of food [9]. Specifically, the lack of access to food in people with disabilities is associated with poverty, which results in higher rates of malnutrition and it is has even been observed that people with disabilities have a higher risk of this condition due to their nutritional requirements [9,10,11]. This problem could further increase the lack of access to adequate quantity and quality of food in the migrant and refugee population with disabilities, leading to an increase in the prevalence of nutritional diseases and worse health outcomes due to their disability status. Although inequalities in access to food is a priority in the Sustainable Development Goals in vulnerable populations (such as people with disabilities) [7], epidemiological studies conducted in LMIC have only focused on the relationship between disability and access to food [12], without considering the migratory status of people in their evaluations.

In Peru, about 10% of the national population has some type of disability [13]. On the other hand, Peru is the second Latin American country that hosts the largest number of Venezuelan migrants since 2016, reaching a figure of more than one million migrants in the last year [13]. In this regard, a previous study conducted in the Venezuelan migrant and refugee population residing in Peru reported that food insecurity in this population exceeded 60% (11% with a severe food insecurity and 52% with moderate food insecurity) [14]. Although studies show high rates of lack of access to food in the migrant and refugee population in Peru, there is little scientific evidence on this outcome in people with disabilities according to their migratory status. In fact, the scarce biomedical literature only focuses on studies that evaluate the disadvantages of people with disabilities in terms of public policies on food and access to health services [15,16]. Therefore, the aim of this study was to determine the association between the presence of disability and the unmet need for access to food in the household of the Venezuelan migrant and refugee population residing in Peru.

## 2. Materials and Methods

### 2.1. Design and Data Source

This study involved a secondary data analysis based on data from the Second Survey of the Venezuelan Population Residing in Peru (ENPOVE 2022—acronym in Spanish) that used a cross-sectional study design to collect the data [17]. The objective of the ENPOVE is to obtain demographic and socioeconomic information on the Venezuelan refugee and migrant population in Peru. The survey was carried out by the National Institute of Statistics and Informatics (INEI—acronym in Spanish) of Peru during the months of February and March 2022. ENPOVE 2022 was sponsored and financed by the World Bank, the United Nations Refugee Agency (UNHCR), the International Organization for Migration (IOM), the United Nations Population Fund (UNFPA), the United Nations Children’s Fund (UNICEF), and the World Food Program (WFP) [17].

The sampling frame was based on information from the Registry of Dwellings and Establishments of the National Labor Market Survey (ENAMEL) and the National Superintendence of Migration of Peru [18]. A sample frame of 236,074 households of the Venezuelan population in Peru was constructed and a total of 195,710 households were obtained, representing 82.9% of the total number of households of the Venezuelan population at the national level. The target population were people of Venezuelan nationality (refugees, refugee applicants, migrants, and people with protection needs), who usually reside in private and collective households in urban areas of the main cities of Peru (Tumbes, Piura, Chiclayo, Trujillo, Chimbote, Ica, Arequipa, Metropolitan Lima) [18]. The ENPOVE 2022 defines a household as a group of people, whether or not they are relatives, who share the main meals and attend to their vital needs together. The information was collected by direct interview and the information was recorded on a tablet by interviewers who were previously trained [18].

The sample selection was probabilistic, stratified, and independent in each study city [18]. The total sample size was 3680 households of the Venezuelan population. A total of 12,487 participants were included in the ENPOVE 2022 database of individuals. Further specifications on the sample design, procedures, and data collection can be found in the ENPOVE 2022 data sheet and report [17,18]. The sample selected for this study consisted of 7739 Venezuelan migrants and refugees aged 18 years or older with complete data on the variables studied. 

### 2.2. Variables

#### 2.2.1. Outcome

Based on the response to the question “What would be the three main needs, which are not being met in your household today?”, we defined the dependent variable of the study as “Unmet need for access to food in the household” being a “Yes” when the participants answered that “Food” would be one of the main needs not being met in the household, and as “No” otherwise.

#### 2.2.2. Exposition

The disability variable was constructed from the Washington Group’s disability questions [19]. The questions were: Do you have permanent limitations in moving or walking, in using arms and legs? Do you have permanent limitations in seeing, even when wearing glasses? Do you have permanent limitations in speaking or communicating, even when using sign language or other? Do you have permanent limitations in hearing, even when using hearing aids? Do you have permanent limitations in understanding or learning (concentrating and remembering)? Do you have permanent limitations in relating to others, because of your thoughts, feelings, emotions, or behaviors? The possible response categories were yes or no. We considered a person to have a disability if they answered yes to at least one of the questions.

#### 2.2.3. Covariates

The following variables were selected as potential confounders: age in age groups (18–59 years, 60 or older), gender (male, female), educational level (up to primary education, secondary education, higher education), chronic disease (no, yes), migration permit (no, yes), work in the last week (no, yes), wealth index (low, middle, high), period of arrival to Peru (pre-pandemic, during the COVID-19 pandemic), and city of residence (Tumbes, Piura, Chiclayo, Trujillo, Chimbote, Ica, Arequipa, Metropolitan Lima).

The creation of the wealth index took into consideration housing characteristics (walls, roofs, floors, water, drainage and lighting) and household goods or services (internet, television, stove, blender, iron, computer, cell phone, landline, radio, refrigerator and washing machine). Each characteristic was recategorized as a dichotomous variable (yes, no), assigning it a score generated through principal component analysis and each household was assigned a score for each characteristic, and the scores for each household were summed [20]. From the results that included the sample weights, households were classified according to the total score of the household, and three equal categories (terciles) were created: “low”, “middle”, and “high”.

### 2.3. Statistical Analysis

Stata 17.0 (StataCorp, College Station, TX, USA) was used to clean, recode, and analyze the data. All analyses included the complex sampling characteristics and sampling weights of the ENPOVE 2022, in addition to the *subpop* command (respondents aged 18 years or older).

Summary statistics and cross tabulations were used to describe the study sample. Chi-square tests with Rao–Scott correction were performed to determine the differences between the proportions of the variables included in the study. Poisson log generalized linear regression models (crude and adjusted for potential confounding variables) were fitted to evaluate the association between disability and food need, reporting prevalence ratios (PR) and 95% confidence intervals (CIs) as measures of association. Finally, a *p* value less than 0.05 was considered statistically significant. 

### 2.4. Ethical Considerations

The ENPOVE 2022 surveyors obtained informed consent from the participants at the beginning of the survey. The ENPOVE 2022 databases do not contain information that would allow respondents to be identified. The ENPOVE 2022 databases and other relevant documents are freely available in the INEI’s microdata repository (https://iinei.inei.gob.pe/microdatos/ (accessed on 26 January 2023)) and no formal ethical clearance was obtained since we conducted a secondary analysis of publicly available data.

## 3. Results

A total of 7739 migrants and refugees from Venezuela were included in the analysis. The mean age was 33.1 years (standard deviation [SD]: 10.5), and more female than male respondents were included (3999 vs. 3740, respectively). The sociodemographic and economic characteristics of both groups (men and women) are shown in Table 1. 

Table 2 shows that the proportion of unmet need for access to food at home was 45.2% (95% CI: 43.0–47.4). Among migrants and refugees without disability, 44.8% reported unmet food need; in those with disability, 61.4% reported unmet food need. The highest proportions of this outcome were found in people aged 60 years or older (52.5%); females (48.0%); in those with a high school education (52.2%); chronic illness (49.4%); without a legal stay permit (52. 2%); who did not work in the week prior to the survey (54.2%); had a low wealth index (54.0%); had migrated during the pandemic (48.5%); and resided in the city of Tumbes (58.2%), Piura (50.6%), or Chimbote (49.4%).

The proportion of disability among Venezuelan migrants and refugees was 2.1%. The highest proportions of disability were found in those 60 years of age or older (8.5%), had up to primary education (4.2%), had a chronic illness (5.0%), had not worked in the week prior to the survey (4.2%), had migrated during the pandemic period (3.0%), and resided in Chiclayo (5.0%) (Table 3).

Regarding the association of interest, the crude analysis showed that there was an association between having a disability and an unmet need for access to food at home (PR: 1.37; 95% CI: 1.17–1.61; *p* < 0.001). This same association was observed in the analysis adjusted for the covariates gender, age, educational level, chronic diseases, work in the last week, wealth index, period to arrival to Peru, and city of residence, with people with disabilities being more likely to have an unmet need for access to food in the household (adjusted PR [aPR]: 1.25; 95% CI: 1.08–1.46; *p* = 0.003) (Table 4).

## 4. Discussion

The objective of this study was to determine the association between the presence of disability and the unsatisfied need for access to food in the households of the Venezuelan migrant and refugee population residing in Peru. It was found that 2 out of 100 Venezuelan migrants and refugees have a disability. On the other hand, almost half of the households in which migrants and refugees from Venezuela reside have an unmet need for access to food. In addition, it was found that migrants and refugees from Venezuela with disabilities were more likely to have an unmet need for access to food at household level.

In relation to the outcome of the present study, approximately 5 out of 10 Venezuelan migrants and refugees in Peru had an unsatisfied need for access to food in their households. This finding is lower than that reported in the official reports conducted in Ecuador and Colombia, in which 82.8% and 85% of households with migrants and refugees from Venezuela had an unmet need for food in 2022 and 2021, respectively [21,22]. These differences could be attributed to the lack of a sampling frame (since they used the databases of non-governmental organizations), the form of telephone interview, and particularly, the Colombian survey was conducted in the context of the COVID-19 pandemic, during which there was a higher proportion of unmet basic needs. These characteristics could increase the proportion of this outcome. Our finding could be attributed to various factors experienced by the Venezuelan migrant and refugee population when they migrate to another country, such as their illegal migratory status, lack of employment, low income, food insecurity, and informality in employment [23,24]. On the other hand, our result reflects a cyclical problem that the population from Venezuela had been experiencing since before their emigration due to the constant lack of adequate food, which could translate into worse nutritional indicators and an increase in diseases or deficiencies.

Regarding the presence of disability, 2% of Venezuelan migrants and refugees reported having a disability at the time of the survey. This finding is in contrast to that reported by the UNHCR and the Latin American Network of Persons with Disabilities and their Families (RIADIS), which estimated that 39.0% of the Venezuelan population with disabilities and their families reside in Peru [4]. This difference could be attributed to the fact that healthier people with greater economic resources are more likely to migrate to other countries [25,26], which could explain the advantage in terms of disability of these migrants compared to other populations. On the other hand, this figure could expose a social and public health problem because people with disabilities who have migrated to other countries experience situations of discrimination, violation of human rights, and unmet basic needs of access to health services and food [4,27,28], which could be reflected in worse living standards for a population that, due to its characteristics, has greater needs. In this sense, the main non-governmental institutions, such as UNHCR, seek to guarantee access to vital services for migrants with disabilities and encourage the application of their skills for their own benefit. Nonetheless, there are still problems of social inclusion of this population due to the lack of access to disability certificates or incorporation of this population in humanitarian programs [4,29,30]. Therefore, strategies for the support and social inclusion of this vulnerable population should be reinforced with the objective of reducing the social and economic inequality they experience due to their migrant and disability status.

As a main finding, our study found that Venezuelan migrants and refugees with disabilities were more likely to have an unmet need for access to food. While the Venezuelan migrant and refugee population already had a lack of food access and availability prior to emigration [8], our finding could be attributed to the impact of emigration coupled with the health and economic crisis caused by the COVID-19 pandemic [4]. Both precedents have generated inadequate living conditions in the migrant and refugee population with disabilities due to the loss of income sources and eviction from their homes, resulting in poor access to basic needs such as food [4,31]. In addition, there is a possibility that given the lack of resources, these households consume hypercaloric and nutrient-poor foods, which according to the biomedical literature, are associated with obesogenic dietary behavior, worse nutritional indicators, and an increase in the incidence of chronic noncommunicable diseases [32].

The public health implications of this study are based on three main considerations. First, intersectoral work should be carried out on migrants and refugees with disabilities to promote their social inclusion, access to basic needs, and legal permanence in the city where they reside. Second, Peruvian governmental institutions should focus on the fulfillment of Sustainable Development Goal 2.1, which seeks to ensure access to all populations, especially people who experience greater vulnerability (such as people with disabilities) [7] to reduce the prevalence and burden of disease due to nutritional diseases. Finally, governmental and non-governmental institutions should redouble their efforts to continue humanitarian support to migrants and refugees with disabilities, which is one of the main recommendations issued by UNHCR and RIADIS, in order to cover basic needs such as employment, access to health services, and food [4].

One of the main strengths of this study is the use of a survey, including a large number of Venezuelan migrants and refugees residing in Peru, that was designed by INEI in conjunction with the main international organizations that seek the protection of migrants and refugees, and thereby provides a current overview of the disability status of this migrant and refugee population. Nevertheless, this study has limitations. First, the lack of temporality in the measurement of the variables due to its cross-sectional design prevents establishing causality. Second, there could be an information bias when considering specific events that occurred in the past. Third, it is possible that there was an over-report or under-report of disabilities by the participants, as previously described [33]. Finally, some variables may not have been included as potential confounders, such as the migrants having food insecurity before moving to Peru.

## 5. Conclusions

In conclusion, it was found that approximately 50% of migrants and refugees from Venezuela have an unmet need for access to food, while 2% have a disability. In addition, having a disability increased the probability of having an unmet need for food access in this Venezuelan population. Considering these findings, non-governmental institutions together with the Peruvian state should focus their strategies on social inclusion and economic and humanitarian support for migrants and refugees with disabilities to improve their access to basic needs such as food, in order to reduce negative nutritional indicators and the occurrence of chronic non-communicable diseases. In addition, our research provides evidence on the main dietary requirements of a minority population that has been understudied and neglected in recent decades.

## Figures and Tables

**Table 1:** Characteristics of the Venezuelan migrants and refugees included in this study, ENPOVE 2022.

Characteristic	Males *n* (%) *	Females *n* (%) *
Overall	3740 (100)	3999 (100)
Age, mean (+/− SD)	33.1 (10.5)	35.0 (12.2)
Age group (years)		
18–59	3633 (97.5)	3807 (95.1)
60 or more	107 (2.5)	192 (4.9)
Educational level		
Higher education	1422 (41.2)	1843 (50.7)
Secondary education	1782 (48.5)	1669 (40.4)
Up to primary education	536 (10.3)	487 (8.9)
Chronic disease		
No	3327 (90.1)	3075 (77.2)
Yes	413 (9.9)	924 (22.8)
Migration permit		
No	1307 (27.2)	1560 (32.1)
Yes	2433 (72.8)	2439 (67.9)
Work in the last week		
Yes	3341 (89.8)	2498 (63.9)
No	399 (10.2)	1501 (36.1)
Wealth index		
Low	1235 (30.2)	1182 (27.2)
Middle	1260 (34.2)	1366 (33.2)
High	1242 (35.6)	1442 (39.7)
Period of arrival to Peru		
During the COVID-19 pandemic	892 (22.1)	1113 (26.0)
Pre-pandemic	2848 (77.9)	2886 (74.0)
City of residence		
Arequipa	215 (3.5)	213 (3.3)
Chiclayo	198 (1.4)	242 (1.6)
Chimbote	258 (1.6)	262 (1.5)
Ica	217 (2.7)	188 (2.2)
Metropolitan Lima	2004 (83.0)	2162 (83.3)
Piura	215 (2.2)	234 (2.2)
Trujillo	410 (4.8)	449 (5.0)
Tumbes	223 (0.9)	249 (1.0)

* The weighting factor and sample specifications of ENPOVE were included. ENPOVE: Encuesta dirigida a la población venezolana que reside en el Perú. SD: standard deviation, CI: confidence interval. Values are the number of participants and percentages, unless stated otherwise.

**Table 2:** Frequency of unmet need for access to food in the household among Venezuelan migrants and refugees by background characteristics, ENPOVE 2022.

Characteristics	Unmet Need for Access to Food in the Household		
	No (*n* = 4242) % * (95% CI)	Yes (*n* = 3497) % * (95% CI)	*p*-Value **
Overall	54.8 (52.6–57.0)	45.2 (43.0–47.4)	
Disability			
No	55.2 (52.9–57.4)	44.8 (42.6–47.1)	0.001
Yes	38.6 (29.6–48.4)	61.4 (51.6–70.4)	
Age group (years)			
18–59	55.1 (52.9–57.3)	44.9 (42.7–47.1)	0.046
60 or more	47.5 (40.2–55.0)	52.5 (45.0–59.8)	
Gender			
Male	57.8 (55.3–60.3)	42.2 (39.7–44.7)	<0.001
Female	52.0 (49.4–54.6)	48.0 (45.4–50.6)	
Educational level			
Higher education	61.8 (58.9–64.5)	38.2 (35.5–41.1)	<0.001
Secondary education	47.8 (44.7–50.8)	52.2 (49.2–55.3)	
Up to primary education	54.0 (48.9–59.1)	46.0 (40.9–51.1)	
Chronic disease			
No	55.7 (53.3–58.0)	44.3 (42.0–46.7)	0.013
Yes	50.6 (46.8–54.4)	49.4 (45.6–53.2)	
Migration permit			
No	47.8 (44.6–51.0)	52.2 (49.0–55.4)	<0.001
Yes	57.8 (55.3–60.2)	42.2 (39.8–44.7)	
Work in the last week			
Yes	57.6 (55.3–59.9)	42.4 (40.1–44.7)	<0.001
No	45.8 (42.5–49.1)	54.2 (50.9–57.5)	
Wealth index			
Low	46.0 (42.4–49.7)	54.0 (50.3–57.6)	<0.001
Middle	51.1 (47.7–54.5)	48.9 (45.5–52.3)	
High	64.9 (61.5–68.1)	35.1 (31.9–38.5)	
Period of arrival to Peru			
During the COVID-19 pandemic	51.5 (48.0–55.0)	48.5 (45.0–52.0)	0.019
Pre-pandemic	55.9 (53.5–58.3)	44.1 (41.7–46.5)	
City of residence			
Arequipa	64.2 (57.1–70.7)	35.8 (29.3–42.9)	<0.001
Chiclayo	55.0 (46.8–62.9)	45.0 (37.1–53.2)	
Chimbote	50.6 (43.8–57.3)	49.4 (42.7–56.2)	
Ica	62.7 (54.5–70.2)	37.3 (29.8–45.5)	
Metropolitan Lima	54.1 (51.5–56.7)	45.9 (43.3–48.5)	
Piura	49.4 (41.6–57.2)	50.6 (42.8–58.4)	
Trujillo	63.1 (57.5–68.3)	36.9 (31.7–42.5)	
Tumbes	41.8 (35.4–48.6)	58.2 (51.4–64.6)	

Data are displayed as weighted % of the row unless indicated otherwise. * The weighting factor and sample specifications of ENPOVE were included. ** Estimated *p*-value using the chi-square test with Rao–Scott adjustment. ENPOVE: Encuesta dirigida a la población venezolana que reside en el Perú. CI: confidence interval.

**Table 3:** Frequency of disability among Venezuelan migrants and refugees by background characteristics, ENPOVE 2022.

Characteristics	Disability		
	No (*n* = 7583) % * (95% CI)	Yes (*n* = 156) % * (95% CI)	*p*-Value **
Overall	97.9 (97.5–98.3)	2.1 (1.7–2.5)	
Age group (years)			
18–59	98.2 (97.7–98.6)	1.8 (1.4–2.3)	<0.001
60 or more	91.5 (87.2–94.4)	8.5 (5.6–12.8)	
Gender			
Male	98.1 (97.5–98.6)	1.9 (1.4–2.5)	0.346
Female	97.8 (97.0–98.3)	2.2 (1.7–3.0)	
Educational level			
Higher education	98.7 (98.1–99.1)	1.3 (0.9–1.9)	<0.001
Secondary education	97.6 (96.8–98.2)	2.4 (1.8–3.2)	
Up to primary education	95.8 (93.5–97.4)	4.2 (2.6–6.5)	
Chronic disease			
No	98.5 (98.0–98.9)	1.5 (1.1–2.0)	<0.001
Yes	95.0 (93.4–96.3)	5.0 (3.7–6.6)	
Migration permit			
No	97.5 (96.6–98.2)	2.5 (1.8–3.4)	0.200
Yes	98.1 (97.6–98.6)	1.9 (1.4–2.4)	
Work in the last week			
Yes	98.6 (98.1–99.0)	1.4 (1.0–1.9)	<0.001
No	95.8 (94.4–96.9)	4.2 (3.1–5.6)	
Wealth index			
Low	98.1 (97.1–98.8)	1.9 (1.2-2.9)	0.542
Middle	98.1 (97.4–98.7)	1.9 (1.3–2.6)	
High	97.7 (96.7–98.3)	2.3 (1.7–3.3)	
Period of arrival to Peru			
During the COVID-19 pandemic	97.0 (95.8–97.9)	3.0 (2.1–4.2)	0.011
Pre-pandemic	98.2 (97.7–98.7)	1.8 (1.3–2.3)	
City of residence			
Arequipa	97.7 (95.6–98.8)	2.3 (1.2-4.4)	0.005
Chiclayo	95.0 (91.6–97.0)	5.0 (3.0–8.4)	
Chimbote	99.1 (97.7–99.6)	0.9 (0.4–2.3)	
Ica	99.7 (98.7–99.9)	0.3 (0.1–1.3)	
Metropolitan Lima	97.9 (97.3–98.3)	2.1 (1.7-2.7)	
Piura	98.7 (96.8–99.5)	1.3 (0.5-3.2)	
Trujillo	98.4 (96.9–99.2)	1.6 (0.8–3.1)	
Tumbes	99.1 (97.0–99.7)	0.9 (0.3–3.0)	

Data are displayed as weighted % of the row unless indicated otherwise. * The weighting factor and sample specifications of ENPOVE were included. ** Estimated *p*-value using the chi-square test with Rao–Scott adjustment. ENPOVE: Encuesta dirigida a la población venezolana que reside en el Perú. CI: confidence interval.

**Table 4:** Association between disability and unmet need for access to food in the household among Venezuelan migrants and refugees, ENPOVE 2022.

Variable	Crude		Adjusted *	
	PR (95% CI)	*p*-Value	aPR (95% CI)	*p*-Value
Disability				
No	Reference		Reference	
Yes	1.37 (1.17–1.61)	<0.001	1.25 (1.08–1.46)	0.003

Weighting factors and sample specifications of ENPOVE were included for all analysis. ENPOVE: Encuesta dirigida a la población venezolana que reside en el Perú. PR: prevalence ratio. aPR: adjusted prevalence ratio. CI: confidence interval. * Model adjusted for gender, age, educational level, chronic disease, work in the last week, wealth index, period of arrival to Peru, and city of residence.

## Data Availability

Not applicable.
